# Maternal Fructose Intake, Programmed Mitochondrial Function and Predisposition to Adult Disease

**DOI:** 10.3390/ijms232012215

**Published:** 2022-10-13

**Authors:** Erin Vanessa LaRae Smith, Rebecca Maree Dyson, Freya Rebecca Weth, Mary Judith Berry, Clint Gray

**Affiliations:** 1 Department of Paediatrics and Child Health, University of Otago, Wellington 6021, New Zealand; 2 Centre for Translational Research, University of Otago, Wellington 6021, New Zealand; 3 Gillies McIndoe Research Institute, 7 Hospital Road, Newtown, Wellington 6021, New Zealand

**Keywords:** fructose, metabolic disease, obesity, intrauterine environment, maternal nutrition, developmental programming, foetal developmental, DOHaD, mitochondrial function, liver metabolism

## Abstract

Fructose consumption is now recognised as a major risk factor in the development of metabolic diseases, such as hyperlipidaemia, diabetes, non-alcoholic fatty liver disease and obesity. In addition to environmental, social, and genetic factors, an unfavourable intrauterine environment is now also recognised as an important factor in the progression of, or susceptibility to, metabolic disease during adulthood. Developmental trajectory in the short term, in response to nutrient restriction or excessive nutrient availability, may promote adaptation that serves to maintain organ functionality necessary for immediate survival and foetal development. Consequently, this may lead to decreased function of organ systems when presented with an unfavourable neonatal, adolescent and/or adult nutritional environment. These early events may exacerbate susceptibility to later-life disease since sub-optimal maternal nutrition increases the risk of non-communicable diseases (NCDs) in future generations. Earlier dietary interventions, implemented in pregnant mothers or those considering pregnancy, may have added benefit. Although, the mechanisms by which maternal diets high in fructose and the vertical transmission of maternal metabolic phenotype may lead to the predisposition to adult disease are poorly understood. In this review, we will discuss the potential contribution of excessive fructose intake during pregnancy and how this may lead to developmental reprogramming of mitochondrial function and predisposition to metabolic disease in offspring.

## 1. Dietary Evolution and Fructose Consumption

Fructose is a 6-carbon polyhydroxy ketone monosaccharide that shares the same chemical formula and is an isomer of glucose [1,2]. When fructose is bound to glucose, it forms the disaccharide sucrose [1,2]. Fructose was a part of the human ancestral diet, in the form of the monosaccharide fructose and disaccharide sucrose, in limited amounts, through seasonal honey, fruit and vegetables [3,4]. Over the last 15,000 years, the change in human behaviour and non-nomadic lifestyles altered how humans survived, without modifying genomic adaptations [5,6]. More recently, from the 1950 to 1960s, techniques to produce high-fructose corn syrup (HFCS) were developed, preceding its introduction into commercialisation in 1967 [7]. HFCS has since remained a beneficial ingredient to commercial food industries because it enhances sweetness and palatability, is stable in acidic products, easy to produce, and unlike sucrose, not susceptible to price and availability [7,8,9,10]. The increased use of refined fructose has dramatically increased fructose consumption from earlier human evolutionary history [3,11,12].

Excess dietary fructose intake per capita has continued to increase worldwide since its introduction to the United States in the early 1970s and remains more prevalent in Westernised countries [4,9,13]. Excess fructose is consumed through a variety of processed foods; however, the highest contributors are sugar-sweetened beverages (SSB) [9,14,15,16]. Current fructose quantities and concentrations consumed in a Westernised diet are difficult to obtain through natural whole food sources; however, they are readily available in nutrient-poor foods and beverages [3,11,12]. Developing countries, such as Mexico, Vietnam, the Philippines, and Indonesia, have shown an increase in demand for HFCS, with exports from the United States escalating from 10-to 100-fold between 2005 and 2012 [17]. However, developed countries, such as the United States, New Zealand, Australia, and many European countries, have all reported stable or declining absolute (g/d) and relative (% energy) added sugar intake over recent years [18]. However, the current amounts of added fructose consumed worldwide are still in excess and significantly above the recommended daily intake levels [17]. Recent data have shown the average caloric intake from added fructose can be between ~10 and 20% [6,19,20] and ~14% in pregnant women [21]. The World Health Organization [WHO] recommends simple sugars from processed foods and SSB should be less than 10% of total daily caloric intake [22]. In perspective, consuming a basal diet of 2000 kcal/day with the addition of one ~16 oz SSB (or ~50–60 g/day) would equate to an intake increase of ~10% of total kcal/day. Despite the relevancy of excess fructose being a significant component in the modern Westernised diet, there remains uncertainty regarding the long-term metabolic health of offspring, where mothers consumed a fructose-rich diet during pregnancy.

## 2. Fructose Metabolism

Following consumption, fructose is absorbed by the small intestine, primarily the jejunum, passing through the brush border of the intestinal wall by glucose transporters [23]. Fructose is transported into the enterocyte by glucose transporter 5 (GLUT5), which is specific to fructose, and through the basolateral pole primarily by glucose transporter 2 (GLUT2) and into systemic circulation via the hepatic portal vein and primarily transported to the liver for metabolism [8,23,24,25]. Additionally, glucose transporter 7 (GLUT7), glucose transporter 8 (GLUT8), glucose transporter 9 (GLUT9) and glucose transporter 11 (GLUT11) have been shown to be fructose transporters, in various degrees of specificity to fructose, due to their sequence homology to glucose transporters [17,25,26,27]. Hepatic fructose uptake from portal circulation is faster than glucose uptake [28] because glucose is not only utilised in the liver but virtually all cells; therefore, glucose is transported from the portal vein to be used by other tissues in the body for energy [29]. Conversely, fructose is minimally used (30–50%) by peripheral tissues, such as kidney, fat, skeletal muscle, brain and testis [4,7]. Moreover, fructose is metabolised differently compared to glucose due to hepatic uptake (50–70%) and rapid conversion into glucose, glycogen, lactate and, more specifically, fat [4,30].

## 3. Hepatocyte Metabolism of Fructose

**Figure 1:**
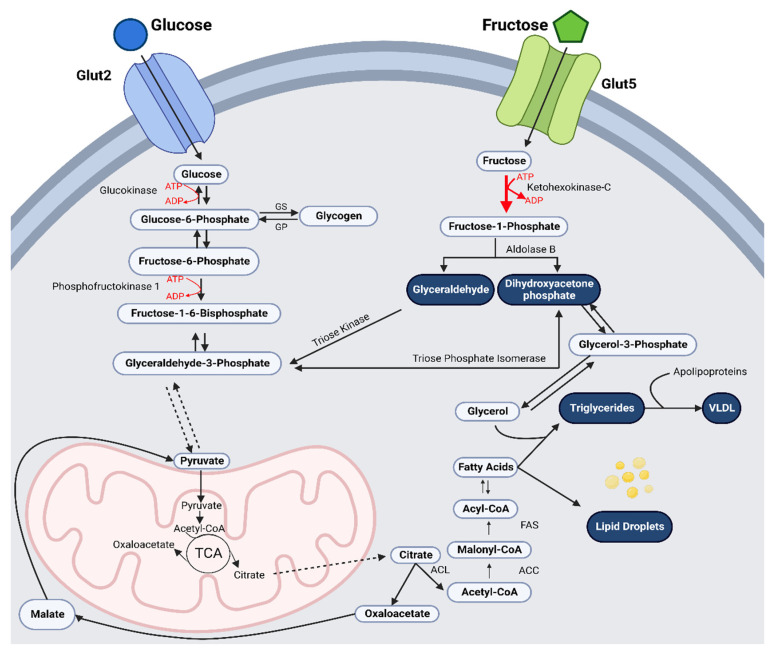
**Fructose Hepatocyte Uptake and Metabolism.** Fructose immediately bypasses phosphofructokinase, a main regulator of glycolysis, and is instantly phosphorylated by fructokinase/ketohexokinase to produce fructose-I-phosphate (fructose-1-P). Aldolase B initiates the lysis of fructose-1-P to produce dihydroxyacetone phosphate (DHAP) and glyceraldehyde. Triose kinase converts glyceraldehyde into glyceraldehyde-3-phosphate (Glyc-3-P). Fructose enters glycolysis at the tri-phosphate level without encountering rate-limiting and regulatory enzymes, providing an unregulated source of both glycerol-3-phosphate (G-3-P) and acetyl-CoA, increasing pyruvate availability to enter the mitochondrion for energy production or hepatic de novo lipogenesis. Primary hepatic metabolites of fructose include glucose, glycogen, lactate, uric acid, free fatty acids (FFA), very low-density lipoproteins (VLDL) and triglycerides (TAG). Diagram created with BioRender.com, accessed on 27 September 2022.

The primary pathway of fructose metabolism occurs through phosphorylation by fructokinase/ketohexokinase. In comparison to glucose, fructose immediately bypasses phosphofructokinase, a main regulator of glycolysis and glucose metabolism, by negative feedback inhibition of intracellular adenosine triphosphate (ATP) and citrate build-up. Fructose is rapidly phosphorylated by fructokinase/ketohexokinase, which is specific to fructose, to produce fructose-I-phosphate (fructose-1-P) [8,29,31,32]. Aldolase B initiates the lysis of fructose-1-P to produce dihydroxyacetone phosphate (DHAP) and glyceraldehyde [8,29,31,32]. Fructose enters glycolysis at the tri-phosphate level, without encountering rate-limiting and regulatory enzymes, providing an unregulated source of both glycerol-3-phosphate (G-3-P) and acetyl-CoA, increasing pyruvate availability to enter the mitochondrion for energy production or hepatic de novo lipogenesis [15]. Primary hepatic metabolites of excessive fructose intake can include glucose, glycogen, lactate, uric acid, free fatty acids (FFA), very low-density lipoproteins (VLDL) and triglycerides (TAG) [8,33] (Figure 1).

Hepatocytes’ specialised ability to rapidly break down fructose is of key importance to support the body’s energy requirements. When consumed with glucose, fructose catalyses glucose uptake and glycogen storage in the liver [34]. When plasma levels of glucose or hepatic glycogen stores are low, fructose can enter the glycolytic pathway at the tri-phosphate level to be used to create glucose, glycogen, or lactate for oxidation in extrahepatic tissues [34]. The rapid increase in fructose breakdown increases metabolite production. Metabolites of FFA, VLDL and TAG are essential for cellular function and provide energy reserves in adipose tissue [35]. Whereas hepatic glucose metabolism is tightly regulated by current energy status, fructose is unregulated, even in the presence of high energy availability [4]. When glucose and glycogen are accessible for energy use, hepatic fructose metabolism favors lipogenesis. Consequently, chronic consumption of excess fructose from sweetened processed foods and SSB increases fructose metabolism, increasing abnormal energy flux and hepatic de novo lipogenesis [14,36], accelerating the release of FFA, TAG, and VLDL into circulation [29,32]. Increased lipogenesis has been the cornerstone of the preceding biochemical reactions induced by excess fructose consumption; however, recent evidence suggests that activation of alternate physiological and signaling pathways may contribute to direct and indirect metabolic dysregulation [8,14,28].

## 4. Excess Fructose and Hepatic De Novo Lipogenesis and Triglyceride Synthesis

Hepatic de novo lipogenesis is the liver’s ability to create new lipids from excess non-lipid substrates (acetyl-CoA, DHAP and G-3-P), primarily from carbohydrates [37,38,39,40]. Hepatic de novo lipogenesis is a highly regulated biosynthetic lipogenic pathway that is responsible for synthesising fatty acids, elongation and desaturation of fatty acids and TAG synthesis [28,40,41]. Within the liver, TAG-VLDL lipid droplets can be stored in the perisinusoidal space (space of Disse) as intra-hepatocellular lipids or exported from sinusoids to be released into circulation and stored as TAG in adipose tissue [4,24,40,41,42]. The lipid droplets stored within hepatocytes are energy reservoirs, which can be used when other energy sources are depleted [43]. In addition, hepatic lipid droplets provide reservoirs of structural elements for cellular membrane synthesis, such as sterols, fatty acids, and phospholipids [43,44]. The diverse functions of lipid droplets allow them to facilitate hepatic homeostasis.

While glucose is a principal substrate for hepatic de novo lipogenesis, fructose favours lipogenesis [28,39]. Through a coordinated sequence of enzymatic reactions, de novo lipogenesis following fructose intake can be initiated quickly through the following pathways: (1) via entrance into glycolysis as Glyc-3-P via the unregulated accumulation of intermediates dihydroxyacetone phosphate (DHAP) and/or glyceraldehyde, thereby increasing the production of acetyl-CoA, and (2) via the more direct entrance of lipogenesis as G-3-P via the unregulated accumulation of intermediate DHAP [37,38,40]. Additionally, fructose availability can also indirectly activate hepatic de novo lipogenesis by increasing conversion of glucose and lactate to form TAG [38]. Following fructose intake, the availability of acetyl-CoA entering the tricarboxylic acid (TCA) cycle increases. TCA intermediates accumulate and are released as citrate into the cytoplasm, where it is converted to acetyl-CoA by ATP-citrate lyase [37,39,44].

Additionally, citrate is an allosteric activator of acetyl-Co-A carboxylase, which converts acetyl-CoA to malonyl-CoA [37,39,44]. Fatty acid synthase uses malonyl-CoA as a substrate to produce palmitate. Palmitate can be elongated to fatty acid stearate by enzyme elongation of long-chain fatty acid protein 6 [45]. The saturated fatty acids, palmitate, and stearate are desaturated by stearoyl-CoA desaturase to become monounsaturated fatty acids, palmitolate and oleate [45]. Following desaturation, monounsaturated fatty acids may be esterified to the glycerophosphate backbone of G-3-P to produce the following additional complex lipids: fatty acids, such as stearic acid, palmitoleic acid and oleic acid, membrane phospholipids, cholesterol esters and TAG [44,46]. G-3-P, fructose’s more direct entrance into de novo lipogenesis, favours esterification of unbound free fatty acids to produce TAG [38,41]. Thereby, for the first step of de novo TAG synthesis, fatty acid acyl-Co-As are added to G-3-P by glycerol-3-phosphate acyltransferase to produce lysophosphatidic acid. 1-acylglcyerol-3-phosphate acyltransferase adds another acyl-CoA to produce phosphatidic acid. Phosphatidic acid is then dephosphorylated by lipin1 to produce 1,2-diacylglycerol. Finally, 1,2-diacylglycerol is esterified to a second acyl-CoA by diacylglycerol acyltransferase to form TAG [40,45]. The TAG produced are packaged with apolipoprotein B 100 by the lipidation of microsomal triglyceride transfer protein and neutral lipids within the endoplasmic reticulum to form VLDL. The TAG-VLDL can either be stored in the liver in the space of Disse, as intra-hepatocellular lipids, or exported from sinusoids and released into blood circulation. Animal and human studies have shown that continued excessive dietary fructose intake increases activity of lipogenic liver enzymes, lipid synthesis and circulating LDL, VLDL, HDL and TAG concentrations in the blood [8,13,33,47,48]. It has been established that dysregulation within de novo lipogenesis can give rise to metabolic diseases such as obesity, type 2 diabetes, and non-alcoholic fatty liver disease (NAFLD) [39].

**Figure 2:**
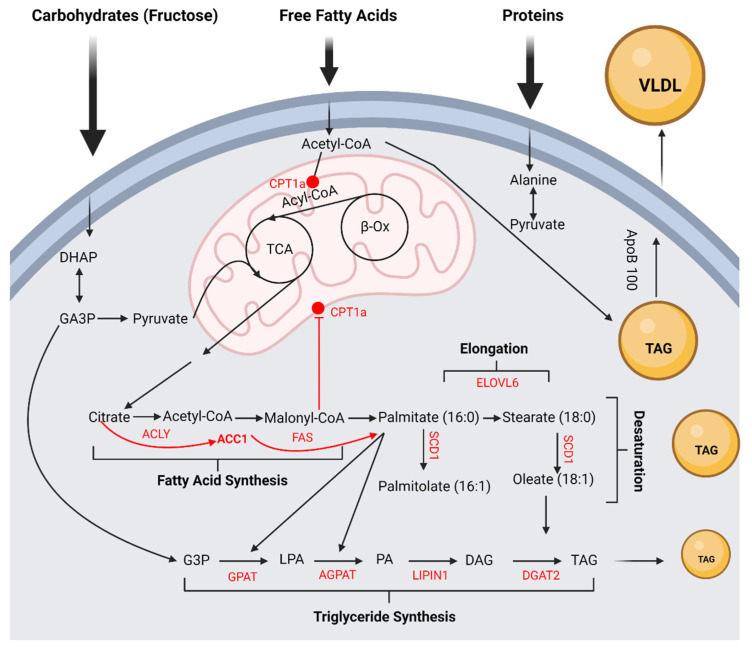
**Fructose and Hepatic De Novo Lipogenesis.** Fructose intake can initiate de novo lipogenesis quickly through the following pathways: (1) via entrance into glycolysis as glyceraldehyde-3-phosphate (Glyc-3-P or GA3P) *via* the unregulated accumulation of intermediates dihydroxyacetone phosphate (DHAP) and/or glyceraldehyde thereby, increasing the production of acetyl-CoA, and (2) via the more direct entrance of lipogeneses as glycerol-3-phosphate (G-3-P) via the unregulated accumulation of intermediate DHAP. In the presence of excess acetyl-CoA, following fructose intake, in the tricarboxylic acid (TCA) cycle, TCA intermediates accumulate and release citrate into the cytoplasm. Citrate is converted by ATP-citrate lyase (ACLY) to acetyl-CoA. Additionally, citrate is an allosteric activator of acetyl-Co-A carboxylase (ACC), which converts acetyl-CoA to malonyl-CoA. Fatty acid synthase (FAS), a biosynthetic enzyme, uses malonyl- CoA as a substrate to produce palmitate (C16:0), the first fatty acid product. Palmitate (C16:0) can be elongated to fatty acid stearate (18:0) or longer fatty acids by enzyme elongation of very long chain fatty acid protein 6 (ELOVL6). The saturated fatty acids, palmitate (C16:0) and stearate (18:0), are desaturated by stearoyl-CoA desaturase (SCD1) to become monounsaturated fatty acids palmitolate (16:1) and oleate (18:1). Following desaturation, monounsaturated fatty acids may be esterified to the glycerophosphate backbone of G-3-P to produce the following additional complex lipids: fatty acids, such as stearic acid, palmitoleic acid and oleic acid, and TAG. Fatty acid acyl-Co-As are added to G-3-P by glycerol-3-phosphate acyltransferase (GPAT) to produce lysophosphatidic acid (LPA). 1-acylglcerol-3-phosphate acyltransferase (AGPAT) adds another acyl-CoA to produce phosphatidic acid (PA). PA is then dephosphorylated by lipin1 (LPIN1) to produce 1,2-diacylglycerol (DAG). Finally, DAG is esterified to a second acyl-CoA by diacylglycerol acyltransferase (DGAT) to form TAG [46,56,57]. The TAGs produced are packaged with apolipoprotein B 100 (apoB100) to form VLDL. Diagram created with BioRender.com, accessed on 27 September 2022.

Excess fructose consumption has been shown to cause adaptive increased activity of the hepatic enzymes involved in fructose uptake into the hepatocyte, metabolism, and de novo lipogenesis of FFA and TAG [24]. Chronic increased activation of hepatic enzymes can, therefore, cause continued metabolic dysregulation, leading to obesity, type 2 diabetes and NAFLD. Continued metabolic dysregulation from increased fructose metabolism can lead to postprandial hypertriglyceridaemia [33], which results in the accumulation of hepatic lipids, plasma VLDL, and visceral adipose deposition [4,33]. Visceral adiposity subsequently further contributes to hepatic TAG accumulation, and the increased portal delivery of FFA to the liver can result in hepatic insulin resistance [33]. Studies in humans and rodents [48,49,50,51,52], dogs [53] and non-human primates [54] have demonstrated that excess fructose or sucrose intake induces hyperlipidaemia more than excessive glucose [55] or a high-fat diet [13] (Figure 2).

## 5. Adverse Effects of Excessive Fructose Intake

To better understand offspring predisposition to metabolic disease following excess maternal fructose exposure *in utero*, it is imperative to understand the direct and indirect dysregulation associated with modern fructose intake. In the modern Westernised diet, fructose intake from natural whole foods has declined, while intake from sweetened processed foods and SSB supersedes levels beneficial for survival and health maintenance [6,58]. The typical daily intake of fructose in Westernised diets has become a major public health concern due to the paralleled increase in metabolic diseases, such as dyslipidaemia, hyperlipidaemia, insulin resistance, visceral adiposity, obesity, diabetes, high blood pressure, cardiovascular disease, and NAFLD [4,13,28,41,47,59,60].

The contribution of fructose in the rise of metabolic diseases has remained controversial, despite the increasing evidence in humans and experimental animal models [28,30]. Results from some animal studies may be conflicting because some models utilise supraphysiological levels of dietary fructose—as high as 30–70% [4,9,47,61]. Some human short-term mechanistic studies have also used levels that exceeded the typical level of dietary fructose, ranging from 25% to 35% and 50% of total caloric intake [55,62,63]. While supraphysiological levels of dietary fructose used in animal and human studies are not comparable to the current amounts consumed, they reinforce the adverse metabolic effects of excess fructose intake. However, the use of animal and human studies that utilise comparable ranges to the average dietary fructose intake still show features of hyperlipidaemia, increased plasma lipid concentrations, increased TAG and VLDL, increased uric acid and dose-dependent weight gain [2,4,47,56,64,65]. Some studies have argued that the rise in metabolic diseases may be associated with excess caloric intake rather than the form or quality of the calories consumed [66,67]. It is critical to keep in mind that the chemical form or ‘quality’ of the calorie may be more metabolically influential than the caloric content itself. For example, even though glucose and fructose are calorically equal (4 kcal/g [29]), there are fundamental differences in their metabolic regulation. The difference in metabolic regulation between glucose and fructose, not caloric content, is the primary implicating factor of fructose’s ability to initiate metabolic dysregulation [4]. Therefore, when considering the impact of fructose on the prevalence of metabolic disease in Westernised countries, several factors appear clear. The pathways that direct fructose’s metabolism are unregulated. In the presence of readily available hepatic glucose and glycogen stores, fructose metabolism favours lipogenesis. Consequently, chronic consumption of excess fructose initiates an adaptive series of synchronised responses with interrelated signalling pathways. These adaptive responses to an abnormal energy flux and hepatic de novo lipogenesis may contribute to direct and indirect metabolic dysregulation [8,14,28].

## 6. Maternal Diet and Predisposition to Metabolic Disease

Suboptimal maternal nutrition and nutrient availability supplied to the developing blastocyst, foetus, neonate, and timing of nutritional ‘insults’ are important in determining the trajectory of foetal development and later-life disease risk. Studies of heredity and maternal environmental influence during pregnancy have been reported since the 1800s [57,68]. However, early epidemiological reports of ‘modern’ developmental programming were published by Kermack et al. in 1934 [69] and were replicated in animal studies that used Shetland ponies [70] and sheep [71]. Furthermore, Susser et al. first published findings on the Dutch Hunger Winter, reporting that men exposed to famine during early foetal life had increased rates of obesity than men exposed during late gestation [72]. David Barker proposed that “Adverse nutrition in early life, including prenatally as measured by birth weight, increased susceptibility to metabolic dysfunction which can include obesity, diabetes, insulin insensitivity, hypertension and hyperlipidaemia, coronary heart disease and stroke” [73]. This concept was coined as the ‘Barker hypothesis’ or the ‘developmental origins of health and disease’ (DOHaD). The hypothesis was born from epidemiological data in Hertfordshire, where he observed that death rates from heart disease correlated with low birth weight, and that some environmental influences during early stages of development appeared to increase cardiovascular disease risk in adult life [73].

Evidence shows that a range of specific macro-, and micronutrient nutritional insults, environmental pollutants and pregnancy complications can affect offspring development, growth, and life-long predisposition to disease. Advancements in methodological technologies have enabled scientists to investigate an ever-growing number of mechanisms involved in developmental programming and have brought about new branches of research, such as the maternal microbiota and mitochondrial function, as key determinants of developmental programming and the predisposition to disease in offspring. Furthermore, the development and refinement of animal models has begun to elucidate molecular aspects of programmed offspring predisposition to later-life disease. These developmentally programmed effects or underlying predisposition to non-communicable diseases may also be amplified by an adverse diet or sedentary lifestyle during later life. However, little is known regarding the potential negative effects of excessive fructose intake during pregnancy and lactation on future offspring’s non-communicable disease risk.

## 7. Excess Maternal Fructose Intake and Offspring Predisposition to Metabolic Dysfunction

Animal models have shown that unbalanced maternal nutrition, in particular, overnutrition, can have permanent effects on the offspring’s organ structure and function, predisposing them to adult-onset of non-communicable diseases, such as obesity, diabetes, NAFLD and cardiovascular risk factors [4,6,74,75]. Contemporary Western women’s consumption of high levels of fructose before and/or during pregnancy and lactation may alter critical phases of pregnancy, such as embryogenesis, foetal-placental development, and milk production and quality [4].

Previous research in rodents has demonstrated maternal consumption of 10% fructose in water during pregnancy and lactation resulted in maternal hyperglycaemia, hyperinsulinaemia, and hypertriglyceridaemia; which were subsequently associated with significantly elevated plasma insulin in the offspring at weaning, suggesting offspring susceptibility to diabetes during adulthood [76]. In animal models where 20% of caloric intake was from fructose during gestation, this resulted in maternal hyperinsulinaemia and sex-specific effects in the offspring, with female offspring having higher plasma leptin and glucose levels and displaying greater vulnerability to metabolic disturbances in neonatal life than male offspring [77]. Our research has shown sex-specific cardio-metabolic differences in the offspring of maternal 10% *w*/*v* fructose-fed rat dams [78]. In addition, a 10% *w*/*v* fructose intake in guinea pigs produced changes in dams’ milk quality, and offspring liver function, lipid metabolism, and programmed hepatic mitochondrial dysfunction [47,79,80].

In our guinea pig model, dams’ dietary intervention of 10% *w*/*v* fructose intake finished immediately following spontaneous delivery of offspring, and consequently, fructose concentrations in dams’ milk were not assessed [47]. However, two studies have shown that during breastfeeding, fructose is transferred from the mother to the infant and that a positive association between breast milk fructose concentration and infant adiposity at 6 months of age can be observed [81,82].

In our study, we were interested in excess maternal fructose intake and dams’ milk FFA composition, and the effects of altered milk composition on the vertical transmission of FFA from dam to offspring [47,79,80]. Research from our lab has shown significantly increased FFA in dams’ maternal milk, including myristic acid, total trans FFA, vaccenic acid, linolelaidic acid, cis-vaccenic acid, total omega-7 and gamma-linolenic acid, the majority of which are trans-fats [47,80]. Other studies have also shown that trans FFA content in lactating mothers is both directly associated with short and long-term maternal diet and independent of diet via maternal adipose tissue [83,84]. Our research has also shown significantly increased FFAs and, specifically, palmitoleic acid and total omega-7 in both mother and offspring from age day 0 to 4 months [41,78,80]. Studies have demonstrated that increases in serum palmitoleic acid indicate a shift of carbon from carbohydrates to FFA and visceral lipid cell lipolysis [79,80,85,86,87]. These results suggest a programming effect on *in utero* exposure to excess fructose on FFA synthesis, fatty acid oxidation and subsequent excess palmitoleic acid.

Similar to our previous studies, others have also reported that a high fructose intake alters β-oxidation, increases FFAs and TAG, causing dyslipidaemia, hepatic lipid accumulation and insulin resistance [14,64,88], and increases in foetal hepatic enzyme activity are associated with fructose uptake, metabolism, lipogenesis and inflammatory response. These early physiological adaptations may potentially predispose offspring to metabolic diseases in later life [2,4,48,89].

In relation to energy availability, insulin signals the hypothalamus to regulate food intake and energy homeostasis. It has been shown that fructose does not stimulate insulin secretion from pancreatic B-cells, most likely due to the low expression of GLUT5 fructose transporters in B-cells, and its effects are, therefore, independent of insulin secretion [90,91,92]. Furthermore, there is strong evidence in animal and human studies that shows that decreases in hepatic and muscle insulin sensitivity are associated with ectopic lipid deposition and tissue-specific lipotoxicity, following long-term excess fructose consumption [30,62]. It is suggested that the increase in hepatic lipids following fructose intake increases the concentrations of diacylglycerol (DAG), which activates protein kinase C epsilon (PKCε), leading to increased serine phosphorylation of the insulin receptor and insulin receptor substrate 1 (IRS-1), resulting in impaired insulin activation [93,94]. Typically, when excess lipids are stored within adipose tissue, adipocytes release the leptin peptide in proportion to the amount of adipose in the body. Leptin signals the hypothalamus when there is enough stored energy by decreasing appetite stimulation and food intake, increasing the sympathetic nervous system to activate the metabolic rate, and decreasing insulin secretion from pancreatic beta cells to decrease energy storage [95]. However, when fructose is consumed in excess, leptin’s feedback signals are perturbed, and as a result, the unregulated over-abundant source of lipid synthesis increases energy availability within adipose tissue. Chronic high concentration of leptin results in leptin resistance, which inhibits satiety signals to the hypothalamus, causing appetite dysregulation by overeating [96].

Ghrelin is an orexigenic peptide hormone produced by endocrine cells in the stomach. It is involved in regulating food intake by stimulating neuropeptide Y and agouti-related protein neurons in the hypothalamus. It acts by decreasing fat oxidation and regulates energy homeostasis [97,98]. Dietary glucose has been shown to suppress ghrelin secretion, in contrast to fructose, which increases ghrelin secretion [99,100]. There is significant evidence that suggests that the consumption of fructose increases circulating ghrelin but has little effect on the secretion of insulin and leptin. This dysregulation may result in impaired intracellular communication of energy availability, contributing to metabolic and appetite dysregulation [96,99,100]. A recent study in rats demonstrated that the maternal fructose-induced dysregulation of satiety signals via ghrelin pathways in the mother was vertically transmitted to the offspring, with both dams and pups having significantly increased ghrelin following maternal fructose intake [101].

Leptin, ghrelin, and insulin-like growth factors are known to represent mediators of appetite and metabolism. Additionally, they play an important role in the brain somatic crosstalk and the complex axis, which controls the gastrointestinal tract and hypothalamic regulation of hunger and satiety. When excess fructose is consumed, circulating leptin decreases, causing increased appetite signals to the hypothalamus [96,99,100]. Recent studies have demonstrated that maternal fructose intake of 10%*w*/*v* increased leptin signalling in the offspring [101,102]. Kisioglu et al. also found that maternal fructose increased offspring leptin levels, despite obesity-related leptin resistance, indicating that fructose intake can affect appetite regulation in offspring from mothers that consumed increased fructose consumption during pregnancy, without the offspring consuming fructose themselves [101].

In context, dysregulation of the insulin–leptin–ghrelin axis may have important consequences for the development of energy homeostasis and appetite regulation in offspring. The long-term effects of excess maternal fructose consumption on offspring appetite regulation are largely unknown. However, it has been shown that fructose in highly processed foods and SSBs can alter hypothalamic response to appetite regulation via the imbalance of leptin and ghrelin secretion in offspring [101,102]. The dysregulated signals of leptin and ghrelin in the hypothalamus stimulate appetite, causing increased consumption and increased lipogenesis. There is an increasing body of literature that highlights a life-long susceptibility to adverse metabolic effects of dietary consumption of fructose in these offspring exposed to high fructose *in utero*. Further studies are needed to better understand the mechanisms underlying the effects of excess maternal fructose through pathways of insulin, leptin, ghrelin, and altered hypothalamic function, which may be important in acutely fed individuals and maternally fructose-exposed offspring.

There is a paucity of data that examine the mechanistic impact of increased fructose intake before and during pregnancy and subsequent adverse effects on lactation, foetal development, and offspring metabolic function. It is essential to determine how the vertical transmission of such deleterious metabolic effects might increase de novo lipogenesis, fatty acid acylation and subsequent metabolic programming in offspring. During foetal development, the liver will undergo many changes in structure and function, which can influence the rate of absorption and metabolism of nutrients received and supply of metabolites in circulation. It has been shown that the combination of increased plasma levels of VLDL and TAG and inhibition of fat oxidation following fructose consumption may lead to increased intracellular lipid accumulation. Animal studies indicate that the foetal liver is highly influenced by the activation of these pathways when exposed to excess maternal fructose intake, making the offspring vulnerable to insulin resistance later in life. In a rat model, HFCS increased insulin resistance in dams; however, free fructose had a greater effect on insulin resistance in pups [103]. It was proposed that the effects of HFCS may stem from the fructose content rather than sucrose, indicating that excess maternal fructose alters the regulation pathways of glucose and insulin and cell function during development.

## 8. Placental Fructose Transport

The placenta plays a critical role in facilitating nutrient, gaseous and waste transport between maternal and foetal circulation, despite all mammalian placentas sharing many processes. There are crucial differences in placental architecture and function amongst different mammalian species [104,105]. The placenta is highly sensitive and responds dynamically to maternal nutritional status and oxygen supply and has been shown to play a major role in the developmental programming of offspring development, due to alterations in structure and function [104,105]. Furthermore, understanding the placental transport and/or metabolism of fructose is vital in understanding the true effects of maternal fructose intake. During ‘normal’ pregnancy, glucose transporter 5 (GLUT5) is a fructose-specific transporter [106,107]. However, the evidence for GLUT5 in the human placenta is limited and there are relatively few studies that have investigated the expression of GLUT5 in the placenta, with studies showing GLUT5 abundance in the placenta [106,107]. Plasma fructose concentrations during ‘normal’ pregnancy and elevated foetal plasma fructose, when compared to the mother at the time of birth, have been shown previously in sheep and humans [108,109,110]. This would suggest that fructose is being actively transported in foetal circulation. These conflicting data may also be a result of the dynamic nature of placental metabolism during gestation. However, the placenta has been shown, in part, to be mediated by diffusion and placental production of fructose [111,112].

Maternal fructose consumption has been previously associated with exacerbation of placental uric acid production, oxidative stress, mitochondrial function, and foetal/placental ratio, as well as reduced placental structural zones [77,113,114,115]. Studies in mice and rats have shown significant effects on foetal/placental ratios and reduced labyrinthine area and increased GLUT5 in maternal livers of fructose-fed animals [116]. We have data to show that the effects of excess maternal fructose (10% *w*/*v*) intake elevate foetal plasma and maternal plasma fructose beyond levels typically observed in control pregnancies at birth [117]. Given the differences in plasma fructose placental structure, increased oxidative stress (and potential mitochondrial dysfunction) has been observed in placentas of fructose-fed mothers and offspring development of metabolic disease. This may offer a potential mechanistic pathway regarding excessive maternal fructose intake and offspring predisposition to metabolic disease. 

## 9. Maternal Fructose and Offspring Predisposition to NAFLD

Fructose metabolism favours hepatic de novo lipogenesis and when excess fructose is consumed, excess lipids can accumulate in the liver, causing metabolic dysregulation and potentially, NAFLD. An individual’s susceptibility and severity of NAFLD can be linked to alterations in liver development and further indirect effects from adiposity and metabolic dysfunction experienced *in utero* [118].

Studies in non-human primates have shown that maternal obesogenic diets stimulate foetal hepatic steatosis with a correlation between maternal and foetal plasma glycerol concentrations and may influence fat accumulation in the foetal liver [118,119]. In mice, maternal Western diet intake showed epigenetic alterations in DNA CpG methylation in peroxisome proliferator-activated receptor alpha (PPARα), fatty acid synthase (FASN), and insulin-induced gene protein, which contributed to increased NAFLD in offspring [120]. Although the consequences of excess maternal fructose on offspring predisposition to NAFLD in adulthood needs further investigation, there is significant evidence that shows that maternal fructose affects hepatic lipogenesis and metabolic development of the offspring, influencing metabolic function across their lifespan. The dietary effects experienced *in utero* that predispose the offspring to inflammation and NAFLD are often referred to as the “first hit,” whereas the “second hit” is a result of the postnatal exposure further exacerbating the developmentally predisposed risk. Mouralidarane et al. showed, in mice studies, that a combination of a maternal and post-weaning obesogenic diet, compared to the control and post-weaning obesogenic diets, resulted in increased Kupffer cells, lipopolysaccharides, reactive oxygen species (ROS) production and hepatic inflammatory cytokines, with subsequent development of NAFLD [121]. D’Alessandro et al. reported that rodent offspring that were fed a sucrose-rich diet had increased adipose tissue weight, dyslipidaemia, increased very low-density lipoprotein secretion, decreased triglyceride clearance, hepatic steatosis, and significant decrease in mitochondrial fatty acid oxidation. However, these effects were more prominent when exposed to both a maternal and post-weaning sucrose-rich diet [122].

## 10. Mitochondrial Function as a Novel Candidate for Developmental Programming

The mitochondria are the maternally inherited ‘powerhouse’ of the cell and can also regulate cellular function, including cell survival and death. The mitochondria are membrane-bound organelles that are present in all nucleated cells. Cellular energy use and storage are allocated by the homeostatic flux of nutrients that are metabolised into energy compounds and transferred by the mitochondria. This process is primarily performed in the mitochondria outer matrix, which metabolises nutrients into substrates that the mitochondria can further metabolise for energy production in the form of ATP. This process takes place in the inner membrane of the mitochondria and is more commonly known as the electron transport chain (ETC). The ETC metabolises substrates supplied by the TCA cycle in-between and across the five key enzymes or complexes (I–V) of the ETC. Using a series of oxidation–reduction reactions, the ECT complexes move electrons across the complexes to create an electron gradient more commonly known as oxidative phosphorylation, which ultimately produces adenosine diphosphate (ADP) and ATP, providing energy for the cell.

The presence of excess TCA cycle products from the metabolism of excess dietary carbohydrates and/or fat has been shown to cause increases in substrate metabolism via the metabolic mitochondrial pathway, which can produce abnormally high ATP levels [123]. Similar mitochondrial activity has been shown by an ATP increase and redox status in oocytes of mice from mothers that were obese during ovulation and pregnancy [124]. Under normal physiological conditions, this may not be of concern. However, when there is readily available intracellular glucose and glycogen, fructose is converted to lipids as VLDL, FFA and TAG. When fatty acids are broken down and enter β-oxidation as a long-chain-acyl-CoA, they produce one acetyl-CoA from one cycle of β-oxidation and then enter the TCA cycle. The NADH and FADH_2_ produced by both β-oxidation and the TCA cycle are used by the electron transport chain to produce ATP. Excess dietary fructose has been known to affect metabolic signalling pathways, namely, β-oxidation, TCA cycle and oxidative phosphorylation (complex I-V) [125,126]. Acetyl Co-A enters the TCA cycle and converts to energy substrates NADH/FADH2 to enter oxidative phosphorylation. Complex I establishes the proton gradient of the electron transport chain as it pumps the H+ ions into the intermediate space and passes the electron to ubiquinone (Q). Complex II serves as an entrance for electrons from FADH_2_ to be donated to Q (now QH_2_) and releases hydrogen into the cytosol. Since complex II is not a proton pump, it does not directly contribute to the proton gradient. Since electrons from FADH_2_ also do not contribute to the proton gradient, it produces fewer ATP than NADH. The presence of excess fructose and the accompanying circulating free fatty acids *in utero* may increase the influx of energy compounds in the foetal hepatic mitochondria, leading to permanent maladaptive mitochondrial function and an increase in circulating FFA and increased hepatic lipid deposition in offspring.

**Figure 3:**
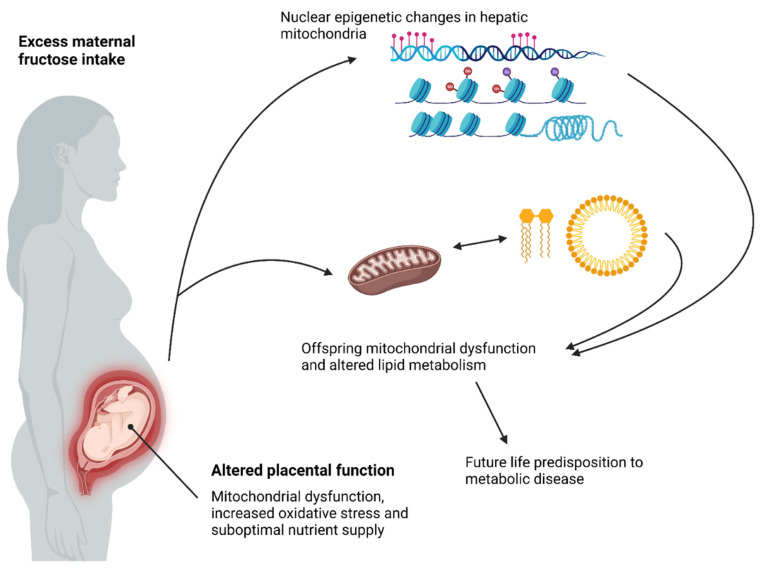
**Proposed mechanisms that influence offspring predisposition to metabolic disease following excess maternal fructose intake**. Proposed mechanisms that influence offspring predisposition to metabolic disease. Excess maternal fructose intake may alter maternal mitochondria function, placental mitochondrial function, increased placental oxidative stress and nutrient supply to the foetus during pregnancy. The subsequent foetal *in utero* environment programmes foetal mitochondrial dysfunction and lipid metabolism via nuclear epigenetic changes in hepatic mitochondria, increased free fatty acid (FFA) production and mechanisms of lipid metabolism. This results in an asymptomatic underlying genotype, which may result in future life predisposition to metabolic disease. Diagram created with BioRender.com, accessed on 27 September 2022.

All mitochondria are maternally inherited due to oocyte cytoplasmic donation to the embryo during early embryogenesis, and paternal mitochondria are killed or die off [127]. A result of this is that all animals carry maternal and paternal nuclear DNA but only maternal mtDNA. Specifically, maternal mtDNA mutations have been shown to play a role in cellular aging, cell death and some cancers [128]. Research into the foetal origins of adult disease and the specific mechanisms underlying these programmed changes following different maternal nutritional insults have been studied extensively. However, most studies are limited to investigating phenotype and organs of interest, with relatively few considering the role of mitochondria. Developmental programming of mitochondrial function and bioenergetics presents a key intracellular candidate, underlying many phenotypes that are observed within the DOHaD paradigm and spread across differing nutritional models of DOHaD. A limited number of studies exist that present evidence of programmed offspring mitochondrial function following gestational nutritional insults or environmental exposures. In a rat model of maternal high fat feeding, Pileggi et al. reported differences in mitochondrial activity in adult offspring skeletal muscle. They showed reduced expression of nuclear respiratory factor-1 (NRF1) and mitochondrial transcription factor-A (mtTFA), as well as a reduction in the genes involved in the ETC and reduction in mitochondrial catalytic activity in ETC complex I and III [129]. Further animal studies have shown links between programmed mitochondrial biology in offspring and increased reactive oxygen species (ROS) production [130,131,132,133,134]. Similarly, although human studies are limited to oocytes from obese mothers, oocytes were shown to have reduced mitochondrial function and impaired oxidative phosphorylation [135]. mtDNA content has been observed to be reduced in placentas from women exposed to environmental stimuli such as smoking, obesity [136,137], stress [136,138], and other environmental pollutants [139,140,141]. A recent review by Gyllenhammer et al. showed that only 31 human studies and 23 animal studies that investigated various gestational exposures and subsequent offspring mitochondrial biology effects had been published [142]. Current research indicates an important role of maternal nutritional status and/or extrinsic environmental factors in the developmental programming of offspring mitochondrial biology [129,142,143]. However, in the context of maternal fructose intake, there are very few published reports that examine the impact of fructose intake before and during pregnancy and the adverse effects on offspring mitochondrial function and how this may be associated with the onset or predisposition to adult disease states (Figure 3). Our laboratory has provided the first example of robust evidence of a significant reduction in VDAC1 expression in weanlings and adulthood in both male and female fructose offspring. Three isoforms of VDAC have been identified, with VDAC1 being the most abundantly expressed. The specific functions of VDAC isoforms are yet to be fully understood. However, they are believed to play a major role in the coupling of cellular energy uptake to mitochondrial ATP production and are involved in the transport of substrates across the outer mitochondrial membrane [144]. VDAC1 is thought to play a major role in controlling metabolic function of the mitochondria for effective energy transfer and efficient ETC function. Huizing et al. have previously shown that a reduction in VDAC1 is detrimental to pyruvate and ATP production [145]. Moreover, further studies have shown that reduced mitochondrial VDAC1 expression causes multiple deficiencies in the ETC and ATP production [79,80].

## 11. Perspectives and Conclusions

Many mechanisms involved in the developmental programming of predisposition to adult health and disease have been postulated. Structural and functional perturbations reported in systemic circulation and organs, including the underlying molecular mechanisms, have been shown to play a role in the development or predisposition to metabolic pathophysiologic conditions. There is now a growing body of scientific literature that demonstrates the importance of maternal environmental stimuli, diet, and the intrauterine environment. These can have an impact upon the development of the preimplantational blastocyst, placenta, and foetus, inducing a predisposition to offspring metabolic dysfunction and progression towards disease during adulthood. The mechanisms involved represent fundamental and important biological processes and can include structural, functional changes and epigenetic modifications that lead to permanent changes in gene and protein expression in key tissues and organs. The relative contribution of these physiological and molecular mechanisms remains unclear. However, there is previously published evidence that indicates that epigenetic modification of mitochondrial genes and subsequent mitochondrial dysfunction may be a key factor in a range of programmed phenotypes in models of developmental programming of health and disease.

The prevalence of metabolic diseases, such as obesity, type 2 diabetes, cardiovascular disease and NAFLD, has increased worldwide over the last three decades [146,147] and are major causes of death in both men and women [148]. Lifestyle factors, such as a Westernised diet and sedentary behaviour, are driving factors of metabolic disease in later life [148]. In particular, the typical daily intake of fructose in Westernised diets has become a major public health concern due to the paralleled increase in metabolic diseases, such as dyslipidaemia, hyperlipidaemia, insulin resistance, visceral adiposity, obesity, diabetes, high blood pressure, cardiovascular disease and NAFLD [8,13,28,41,47,59,60]. In addition to environmental, socioeconomic, and genetic factors, the intrauterine environment is now recognised as a principal factor that contributes to establishing physiological set-points, leading towards a potential susceptibility or predisposition of metabolic disease throughout life [149,150,151].

Research has shown that excess maternal fructose causes developmental programming of altered liver function, including increased de novo lipogenesis, increased mitochondrial dysfunction and programmed changes to ETC complexes, which may underpin this dysfunctional metabolic phenotype [79]. We would further hypothesise that increased fructose exposure during *in utero* development and vulnerable periods for hepatic mitochondrial development may adversely affect the hepatic developmental processes by epigenetic regulation, permanently ‘programming’ mitochondrial function. In particular, altered ETC function, increased ROS and excessive ATP production, and potentially ROS levels, will affect lifelong lipid synthesis and metabolism in offspring.

**Figure 4:**
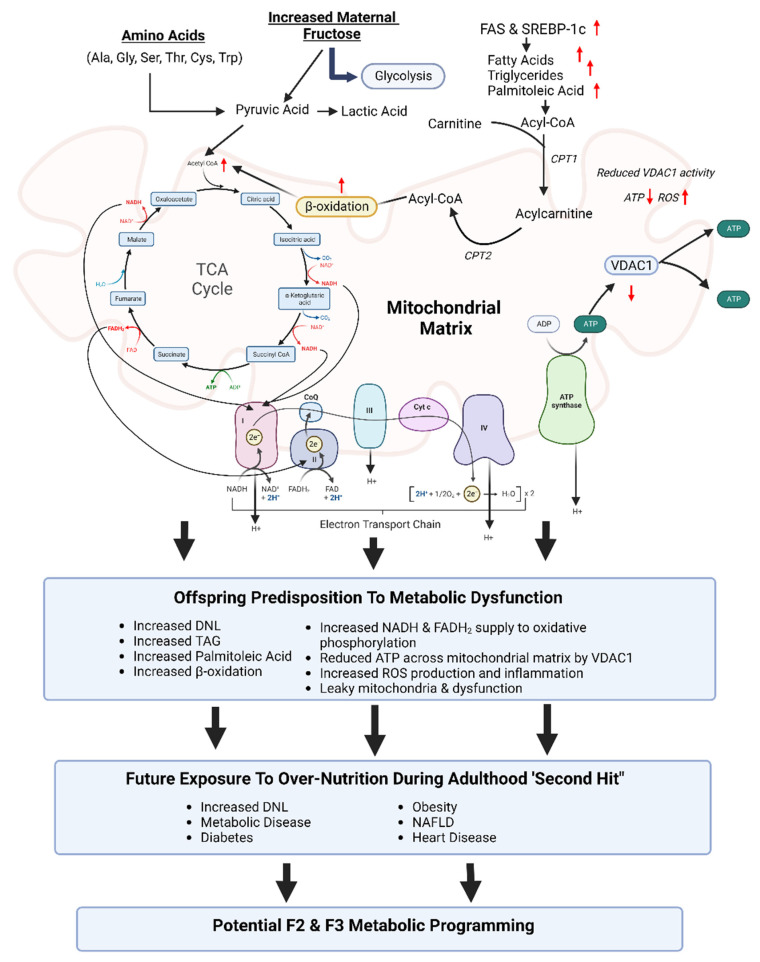
**Proposed developmentally programmed mechanisms of long-term mitochondrial function, following excess maternal fructose intake.** Red arrows indicates offspring metabolic alterations due to excess maternal fructose intake, and the proposed developmentally programmed mechanisms of long-term mitochondrial function and subsequent predisposition to metabolic dysfunction. Following exposure to a ‘second hit’ of over-nutrition during adulthood, this increases the risk of experiencing metabolic disease(s). These programmed mitochondrial effects in F1 may affect germline modifications of F1 foetal gametes, potentially programming F2 and F3 metabolic function. Diagram created with BioRender.com, accessed on 27 September 2022.

We have previously reported, in both male and female fructose offspring, consistent increases in mitochondrial oxidative phosphorylation complexes II and IV and FAS at both weanling and adult ages. However, because the pathways of de novo lipogenesis, β-oxidation and oxidative phosphorylation are intimately linked, it is a classic ‘chicken or the egg’ scenario—are the programmed changes in FAS contributing to increased activation of TCA cycle and β-oxidation, subsequently increasing complex II and IV protein expression; or are the programmed changes in mitochondrial nDNA in complex II and IV driving an altered proton gradient and decrease in ATP movement across the mitochondria matrix by VDAC1, causing mitochondrial dysfunction; or a combination of both? Mitochondrial control of metabolic signalling pathways may be an epigenetically programmed effect of mitochondrial genes, including the ETC subunits of mitochondrial proteins encoded by nuclear DNA (nDNA) genes. All four subunits of complex II are encoded by nDNA, while complexes I, III and IV are encoded by nDNA and mtDNA, the majority of which are encoded by nDNA [152]. Although, the mitochondrial genome (mtDNA) does not contain histones and, therefore, is not believed to be epigenetically regulated [153], when compared to nDNA, which can be more readily epigenetically programmed. Overall mitochondrial function plays a critical role in directing nDNA gene expression via epigenetic modifications, which in turn greatly affects mitochondrial function. In conclusion, this review adds to the current narrative based on previously published data from our own laboratory, demonstrating the detrimental effects of maternal fructose intake on offspring physiology and the asymptomatic molecular phenotype that predisposes offspring to hepatic metabolic dysregulation throughout life (Figure 4). Our recent study on the hepatic proteome shows clear evidence of the *in utero* programming effects of excess maternal fructose intake on offspring mitochondrial function, de novo lipogenesis, and associated increases in key proteins (FAS and SREBP1-c), increased TAG production, and programmed increases in circulating palmitoleic acid throughout life. Taken together, this research and data from other labs may assist in the understanding of how excess maternal fructose influences offspring mechanistic pathways, predisposition to metabolic dysfunction in offspring, and future life health status.
